# The Sierra Platinum Service for generating peak-calls for replicated ChIP-seq experiments

**DOI:** 10.1186/s13104-018-3633-x

**Published:** 2018-07-28

**Authors:** Daniel Wiegreffe, Lydia Müller, Jens Steuck, Dirk Zeckzer, Peter F. Stadler

**Affiliations:** 1 Image and Signal Processing Group, Department of Computer Science, University of Leipzig, Augustusplatz 10, 04109 Leipzig, Germany; 2 Natural Language Processing Department, Department of Computer Science, University of Leipzig, Augustusplatz 10, 04109 Leipzig, Germany; 3 Bioinformatics Group, Department of Computer Science, University of Leipzig, Härtelstraße 16-18, 04107 Leipzig, Germany; 4 Interdisciplinary Center for Bioinformatics, University of Leipzig, Härtelstraße 16-18, 04107 Leipzig, Germany; 5 Max Planck Institute MIS, Inselstraße 22, 04103 Leipzig, Germany; 6 Fraunhofer Institute for Cell Therapy and Immunology IZI, Perlickstraße 1, 04103 Leipzig, Germany; 7 Institute for Theoretical Chemistry, University of Vienna, Währinger Straße 17, 1090 Vienna, Austria; 8 Center for Non-coding RNA in Technology and Health, University of Copenhagen, Grønnegårdsvej 3, 1870 Copenhagen, Denmark; 9 The Santa Fe Institute, 1399 Hyde Park Road, 87501 Santa Fe, NM USA

**Keywords:** ChIP-seq, Peak-caller, Histone modifications, Replicate analysis

## Abstract

**Objective:**

Sierra Platinum is a fast and robust peak-caller for replicated ChIP-seq experiments with visual quality-control and -steering. The required computing resources are optimized but still may exceed the resources available to researchers at biological research institutes.

**Results:**

Sierra Platinum Service provides the full functionality of Sierra Platinum: using a web interface, a new instance of the service can be generated. Then experimental data is uploaded and the computation of the peaks is started. Upon completion, the results can be inspected interactively and then downloaded for further analysis, at which point the service terminates.

## Introduction

ChIP-seq has become an important high throughput technique for analyzing protein–DNA interaction. It is routinely employed for identifying transcription factor binding sites and for determining chromatin states by virtue of immunoprecipitation of nucleosomes that exhibit histones with specific chemical modifications. The basic principle of ChIP-seq is the specific enrichment of immunoprecipitated DNA-protein aggregates, from which—after sequencing the DNA component—the genomic location of the DNA–protein interactions of interest are inferred. The key step in the analysis of ChIP-seq data is peak-calling, that is, the determination of those genomic regions in which immunoprecipitated DNA is significantly enriched relative to “empty” control samples [1].

Since the introduction of ChIP-seq, several peak callers were published, e.g., MACS [2], PeakSeq [3], and csaw [4]. However, most of them handle only one pair of experiment and control or perform a differential peak-calling between two experiments. Their performances were reviewed by Wilbanks and Facciotti [5] and by Koohy et al. [6]. Tools for multi-replicate peak-calling were developed only recently. Among them, Sierra Platinum [7] combines multiple ChIP-seq experiments in a single peak calling process and thus makes full use of the information supplied by replicates. It provides extensive visualization options to guide the user through the evaluation of the experiments. This enables the user to inspect the replicate’s quality in various ways, and to enhance the peak-calling quality by weighting or excluding individual samples. The GUI of Sierra Platinum further facilitates immediately comparing the impact of different parameter settings. Compared to other currently available peak-calling tools, Sierra Platinum performs best with respect to recall and false discovery rate regardless of the data quality [7].

The computational efforts for ChIP-seq analysis require hardware that may not be available in labs without dedicated bioinformatics infrastructure due to the size of the input files and the complexity of the algorithms that combine multiple samples. To overcome this limitation, Sierra Platinum Service provides access to the full functionality of Sierra Platinum by providing a web-based service hosted at sierra.sca-ds.de. In addition, we provide a convenient docker image for easy deployment of private instances of the service, e.g., for institution-wide use.

## Main text

### Methods

The Sierra Platinum Service is a publicly available web service that combines user management, job control, and a queuing system as well as mechanisms for uploading the input data and for downloading all results. It creates a dedicated Sierra Platinum Server that allows the user to upload, analyze, inspect, and manipulate his ChIP-seq data using the Sierra Platinum Client with very little local resource consumption. Finally, the user can download all results—analysis results as well as the final peaks.

#### Usage and interaction

**Fig. 1:**
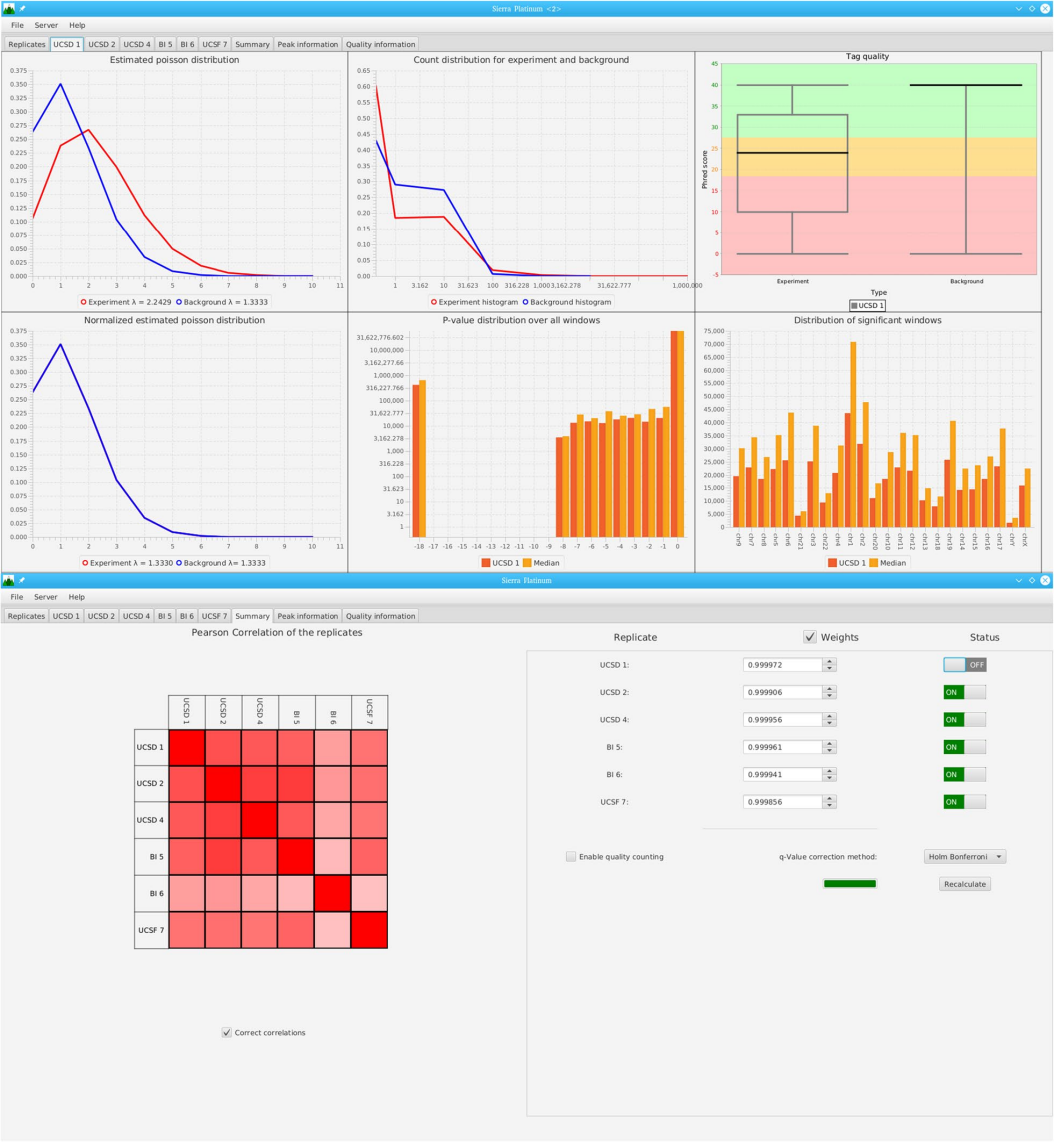
Upper part: A replicate tab in the GUI of SPS. It shows the calculated properties of a replicate and enables the user to assess the quality and validity of the replicate. Lower part: The summary tab in the GUI of SPS. On the left hand side the correlations between the replicates used are displayed. On the right hand side the weighting mechanism controlling the influence the effect of single replicates on the final peak calling result is shown

The service requires registration with a valid email address and allows the user to start a dedicated Sierra Platinum Server (SPS) for which he received the necessary credentials by email. A SPS runs for 72 h or until termination by the user. During this time, the user may disconnect from and reconnect to the server at any time. At the end of the SPS’s life time, all data is deleted from the server hardware. To use the SPS, the user connects with his credentials through the Sierra Platinum Client and first uploads his data as bam files using the integrated FTPS client. Then, the peak calling can be started. Afterwards, quality control information can be visually inspected (see Fig. 1) and parameters may be adjusted as for any local installation of Sierra Platinum (see Müller et al. [7] for details). At any time, the results file can be downloaded for further, local analysis.

**Fig. 2:**
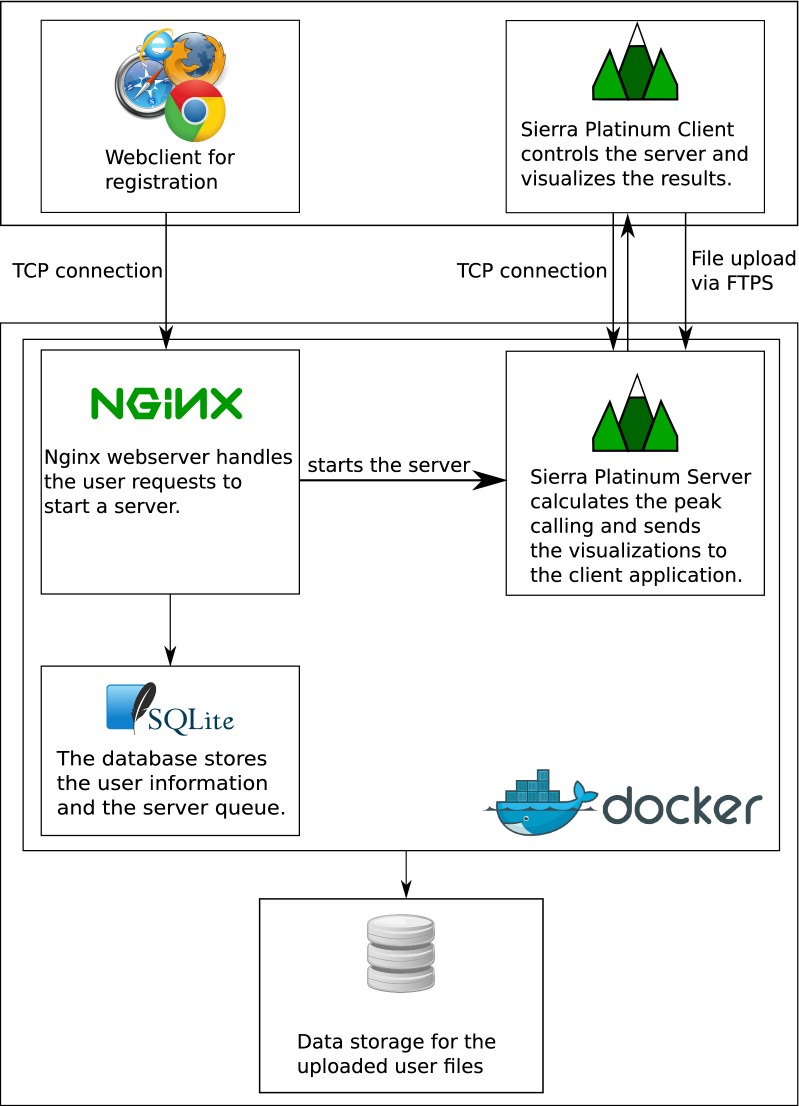
Overview of the Sierra Platinum Service Docker container. The web server handles the user registrations and starts the Sierra Platinum Server after a valid activation. All necessary user information are stored in an SQLite database. After a successful registration the user can upload his data with the Sierra Platinum Client and start the computation. Afterwards, it is possible to export all results and visualizations within the client. The logos of NGINX, SQLite, and Docker were taken from Wikimedia Commons. The Docker and Sierra Platinum logo are licensed under Apache License 2.0

### Technical realization

The server is hosted within a docker container (see Fig. 2), which provides a Java SDK for the SPS, a fully configured nginx web server with php5 support, and an SQLite database that stores the user management of the service. The mail transmission is implemented by using sSMTP that allows using an existing mail address without the need to setup an email server within the service.

Since the Sierra Platinum Service is embedded in a docker container, it can easily be deployed by pulling the Git repository https://github.com/sierraplatinum/sierra-service and running the scripts build.sh for building the container and run.sh for starting it. At this stage, the service can be configured by specifying TCP ports, the email address, and resource limitations such as the number of concurrent SPS instances or threads. To handle the limited number of SPS instances, a queuing system was implemented to handle all user requests. Within the docker container all services start automatically. The upload mechanism was implemented in the client/server core. To address security concerns, every user of the service is assigned his own FTPS directory and is jailed to it.

The client checks the validity of the uploaded files and the server can compute missing .bam indices. Interrupted uploads can be continued on the fly to accommodate for the large size of the input files.

### Conclusion

Sierra Platinum Service provides full access to a state of the art ChIP-seq peak caller that can handle multiple replicates and that features extensive interactive quality control monitoring. Conceived as a server–client structure, it overcomes the need of extensive local computational resources and provides the user with simple client-based access. It is available as a docker container and thus can be deployed easily with little need for configuration, since docker is available for all common operating systems and the configuration of the docker container does not rely on the host system. This facilitates providing dedicated instances at the institutional level. Furthermore, the docker architecture is easily maintainable, since all updates can be pulled from the docker repository. Additionally, the implemented queuing system enables providing a Sierra Platinum Service for a larger group of users.

## Limitations

The SPS architecture is currently designed as a split infrastructure. The registration and validation of a new user is handled by a web interface whereas the data upload, data processing, and data presentation is implemented within a Java GUI application. Therefore, the user needs an up-to-date Java installation on his client. Moreover, the data upload of the input files can take a long time depending on the user’s internet connection and the input size. If the user has a very slow upload rate, the maximal runtime of the service may exceed before finishing the upload. Further, the user needs a valid email address for the registration process.

Currently, the SPS instance at sierra.sca-ds.de is able to compute five jobs simultaneously. If necessary, the service can be extended easily with more slots since it is running on cluster system.

## Abbreviations

SPS: Sierra Platinum Service
ChIP-seq: chromatin immunoprecipitation sequencing
DNA: deoxyribonucleic acid
TCP: Transmission Control Protocol
FTPS: FTP over SSL
